# Platelet Function Tests and Inflammatory Markers for the Differentiation of Primary Thrombocytosis and Secondary Thrombocytosis

**DOI:** 10.31557/APJCP.2019.20.7.2079

**Published:** 2019

**Authors:** Piyapong Kanya, Ekarat Rattarittamrong, Ornkamon Wongtagan, Thanawat Rattanathammethee, Chatree Chai-Adisaksopha, Adisak Tantiworawit, Lalita Norasetthada

**Affiliations:** 1 *Department of Internal Medicine, Chiang Rai Prachanukroh Hospital,*; 2 *Division of Hematology, Department of Internal Medicine, Faculty of Medicine, Chiang Mai University, Chiang Rai, Thailand.*

**Keywords:** Light transmission platelet aggregometry- erythrocyte sedimentation rate- C-reactive protein

## Abstract

**Background:**

The prognosis and management of primary thrombocytosis (PT) and secondary thrombocytosis (ST) are different. This study aims to evaluate the role of platelet function tests by light transmission platelet aggregometry (LTA), plasma von Willebrand factor antigen (vWF:Ag), ristocetin cofactor activity (vWF:RCo) and inflammatory markers [erythrocyte sedimentation rate (ESR), C-reactive protein (CRP)] for the differentiation between PT and ST.

**Methods:**

This prospective study was carried out in patients with platelet counts greater than 450 x 109/L. Primary outcomes were the sensitivity and specificity of platelet function tests by LTA for the differentiation of PT and ST. Secondary outcomes were sensitivity and specificity of ESR, CRP, vWF:Ag, and vWF:RCo for the differentiation of PT and ST.

**Results:**

Fifty-two patients were enrolled onto the study of which 26 (50%) had PT. The sensitivity and specificity of epinephrine, collagen, and arachidonic acid (AA) induced abnormal LTA for the differentiation of PT from ST were sensitivity of 50%, 38.5%, 26.9% and specificity of 88.5%, 100%, 100% respectively. The sensitivity and specificity of abnormal ESR, CRP, and either abnormal ESR or CRP in the differentiation of ST from PT were sensitivity of 88.5%, 80.8%, 100% and specificity of 65.4%, 61.5%, 46.2% respectively. The sensitivity and specificity of low vWF:Ag and vWF:RCo in the differentiation of PT from ST were sensitivity of 7.69%, 42.3% and specificity of 100%, 88.5% respectively.

**Conclusions:**

Abnormal platelet function determined by LTA with collagen, AA, epinephrine had high specificity ratings enabling the differentiation between PT and ST. vWF:RCo, ESR and CRP levels could be helpful in differentiating between PT and ST.

## Introduction

Thrombocytosis is defined as the presence of a high platelet count,specifically more than the upper limit of normal or more than 450 x 109/L. This condition can be classified as primary thrombocytosis (PT) resulting from a clonal bone marrow abnormality such as myeloproliferative neoplasm (MPN), reactive or secondary thrombocytosis (ST) and inherited thrombocytosis(Rodgers et al., 2014; Beer et al., 2011).

The most common form of thrombocytosis is ST. The most common cause of PT is essential thrombocythemia (ET) (Schafer, 2004; Rose et al., 2012). ET, polycythemia vera(PV), primary myelofibrosis(PMF), and chronic myeloid leukemia (CML) are MPNs characterized by clonal proliferation of hematopoietic stem cells. Platelets in these disorders show not only quantitative but also qualitative changes by switching from resting to pro-coagulant phenotypes (Barbui et al., 2013). In addition, a decrease in the number of largevon Willebrand factor (vWF) multimers has been noted in patients with MPN, and this decline correlates with an increased platelet count (Tiede et al., 2011; Kumaret al., 2002).

The natural history of a MPN is marked by thrombohemorrhagic complications and a propensity for the transformation to myelofibrosis and acute leukemia (Carobbio, et al., 2011). In contrast, ST is usually considered as a reactive process without thrombohemorrhagic issues, but its underlying causes must be identified and treated (Schafer, 2004).

The mechanisms of an increase number of platelets in ST are unclear. In many cases, ST is driven by elevated endogenous levels of interleukin (IL) - 6, other cytokines, or catecholamines that may accompany inflammatory, infectious, malignant or stress linked situations (Schafer, 2001). Many markers of the acute phase reactants including C-reactive protein (CRP) and erythrocyte sedimentation rate (ESR) are also significantly elevated in patients with reactive as opposed to clonal thrombocytosis (Bleeker and Hogan, 2011; Bashir et al., 2014; Tefferi et al., 1994).

Platelet function is generally analyzed using a platelet function analyzer (PFA)-100 and light transmission platelet aggregometry (LTA). LTA measures the ability of various agonists to induce in vitro platelet activation (Harrison, 2005). In a previous study into platelet function tests in thrombocytosis, abnormal results are suggestive of ET while normal outcomes from both investigations can be used to exclude ET (Tsantes et al., 2010).

Since data is limited regarding surrogate markers to differentiate PT from ST, the aim of this study was to evaluate the role of platelet functiontests by LTA, plasma vWFantigen (vWF:Ag), ristocetin cofactor activity (vWF:RCo) and inflammatory markers (ESR, CRP)to enable differentiation between PT and ST.

## Materials and Methods

*Study overview*

This was a prospective study enrolling patients aged18 years or older who had platelet counts of more than 450x109/L and were admitted to or attended the out-patient clinic at Chiang Mai University Hospital, Thailand from November 2016 to December 2017. They were categorized as PT based on the revised 2016 World Health Organization criteria (Arber et al., 2016) and ST. All patients in PT group had to have been confirmed by a positive Philadelphia chromosome (for diagnosis of CML), *JAK2*V617F or calreticulin (*CALR*) gene mutation (for diagnosis of PV, ET, or PMF). The patients with ST had to have been diagnosed with underlying diseases which can cause thrombocytosis. Exclusion criteria were patients had been given chemotherapy agents or a tyrosine kinase inhibitor within four weeks prior to evaluation and/ or received antiplatelet or non-steroidal anti-inflammatory drugs within 14 days prior to evaluation, or had chronic kidney disease with a decreased glomerular filtration rate less than 60 ml/min/1.73 m2 for more than three months. Written informed consent was obtained from all patients. Blood samples were obtained sent for LTA, ESR, CRP, vWF:Ag, and vWF:RCo analysis. Controlled blood samples for LTA were obtained from volunteers.The agonists in the platelet function test by LTA (Chrono-Log Aggregometer Model 700, United States) were high dose adenosine diphosphate (ADP) 10 micromolar (µM), epinephrine (Epi) 10 µM, high dose ristocetin 1 mg/mL, arachidonic acid (AA)1 millimolar (mM)and collagen 2 µM. Abnormal LTA was defined as platelet aggregation less than 60% at five minutes after adding the agonists.

Whole blood samples anti-coagulated with EDTA were used to determine ESR level based on red blood cell aggregation method (Ves-Matic Cube 30, Italy). The CRP level was tested using monoclonal antibodies specific to human CRP, aggregated when mixed with plasma containing CRP (BN ProSpec system, United States). Abnormal ESR and CRP were defined as values greater than the upper limit of normal (> 10 mm/hr and > 5 mg/L respectively). The level of vWF:Ag was measured by an automated latex enhanced immunoassay (ACL TOP 500, United States). The vWF:RCo was tested by measuring the ability of a vWF in the plasma which causes an agglutination of the ristocetin and stabilizes platelets contained in the von Willebrand reagent (Sysmex CS-2500, Japan). Low vWF:Ag and vWF:RCo was defined as a value less than the lower limits of normal (vWF:Ag< 42% in blood group O and < 66% in other blood groups; vWF:RCo< 70%). Clinical characteristics and thrombohemorrhagic complications were assessed from the patients and medical records.

*Outcomes*

Primary outcomes were the sensitivity (Se) and specificity (Sp) of platelet function tests by LTA enabling the differentiation between PT and ST. Secondary outcomes were Se and Sp of ESR, CRP, vWF:Ag, and vWF:RCo for the differentiation of PT and ST as well as correlations between these markers and thrombohemorrhagic complications in patients with PT and ST.

*Statistical Analysis*

Descriptive statistics were presented as a mean with corresponding standard deviation (SD), a median with range, or percentages.Categorical variables were compared using the Chi-squared test or Fisher exact test as appropriate. Continuous variables were compared using a student t-test or Wilcoxon rank-sum test (Mann-Whitney test) as appropriate. The platelet function tests and inflammatory markers for the differentiation of ST and PT were presented as Se, Sp, positive predictive value (PPV), and negative predictive value (NPV). The factors associated with thrombohemorrhagic complications were presented as odds ratio (OR) and 95% confidence interval (CI). Level of statistical significance was defined as p-value < 0.05. For a power of 95% and α error of 0.05 to detect differentiation of primary outcome between groups, abnormal results of platelet function test were estimated at 80% in cases of PT and 30% in ST (Tsantes et al., 2010). The total number of patients needed for enrollment was calculated to be 46. Analyses were performed using the Statistical Package for Social Sciences,version 22.0.

## Results

*Clinical Characteristics of Patients*

Fifty-two patients with a median age of 49.5 (range, 18 - 89) years were included in the study. Twenty-three patients (44.2%) were male. There was no significant difference in age and sex between groups. Twenty-six patients (50%)had PT including 14 (53.8%)CML, nine (34.6%) *JAK2 *V617F mutated ET, one (3.9 %) *CALR* mutated ET, one (3.9%) *JAK2*V617F mutated PMF, and one (3.9%) *JAK2*V617F mutated PV. Twenty-six patients (50%) had ST including ten (38.5%) malignancies, eight (30.8%) chronic infections, five (19.2%) post-splenectomy states, and three (11.5%) autoimmune diseases. In this group, twenty-one patients (80.8%) had a normal platelet count when followed up in the outpatient clinic. The rest of the patients that had a persistent thrombocytosis were secondary from post-splenectomy. Seven patients (26.9%) in the ST group also underwent *JAK2 *V617F testing, all tested negative.

**Table 1:** Clinical Characteristics of Patients with Primary and Secondary Thrombocytosis

Parameters	Total (N = 52) mean ± SD	PT (N = 26) mean ± SD	ST (N = 26) mean ± SD	P-value
Age (years)	51.1 ± 18.2	52.6 ± 19.6	49.5 ± 16.9	0.547
Hb (g/dl),	9.9 ± 2.3	10.4 ± 2.8	9.5 ± 1.6	0.163
WBC (x 109/L),	16	61	13.1	< 0.001
Platelet count (x 109/L),	928.6 ± 491.9	1,149.5 ± 593.2	707.6 ± 200.9	< 0.001

Hb, hemoglobin; WBC, white blood cell; SD, standard deviation; PT, primary thrombocytosis; ST, secondary thrombocytosis; JAK2, Janus Kinase 2; *CALR*, calreticulin; SD, standard deviation; min, minimum; max, maximum.

No significant difference was noted in the mean hemoglobin (Hb) level between groups. The median white blood cell (WBC) count was greater in the PT patients compared with ST (61 x 109/L vs. 13.1 x 109/L, p < 0.001). The mean platelet count was also greater in PT patients compared with ST (1,149.5 x109/L vs. 707.6 x109/L, p < 0.001). According to the clinical presentation reports, fever was more common in the ST patients compared with PT (57.7% vs. 11.5%, p < 0.001). In contrast, splenomegaly was more common in cases of PT compared with ST (69.2% vs. 7.7%, p < 0.001) (Table 1 and 2). In the ST group, all patients with splenomegaly were diagnosed with lymphoma.

Comparison of frequency of parameters such as platelet function tests using light transmission platelet aggregometry, inflammatory markers, von Willebrand factor antigens, ristocetinco factor activity and thrombohemorrhagic complications between primary and secondary thrombocytosis is shown in Table 3.

*Platelet Function Tests in Primary and Secondary Thrombocytosis*

The median platelet aggregation in response to Epi was significantly decreased in the PT patients compared with those with ST (60% vs. 74.5%, p = 0.012) while there was no significant difference observed in response to ADP, collagen, ristocetin and AA (Table 4). The proportion of the patients with abnormal LTA induced by Epi, collagen and AA were significant higher in PT patients compared with ST.

*Inflammatory Markers in Primary and Secondary Thrombocytosis*

Median ESR (8.4 mm/hr vs. 57 mm/hr, p < 0.001) and CRP (3.8 mg/L vs. 27.1 mg/L, p < 0.01) were significantly lower in PT patients compared with ST (Table 4). The proportion of patients with high abnormal ESR and CRP levels were significantly lower in PT patients compared with ST. There was a significant higher proportion of patients in ST group who has abnormal levels of both ESR and CRP as compared to PT group.

*vWF:Ag and vWF:RCo in Primary and Secondary Thrombocytosis*

Mean vWF:Ag (150.8% vs. 210%, p = 0.015)

and vWF:RCo (90.5% vs. 148.3%, p < 0.001) were significantly lower in PT patients compared with ST (Table 2). No significant difference in proportion of patients with low vWF:Ag was shown between groups whereas the PT group had a significantly greater proportion of patients with low vWF:RCo.

*Thrombohemorrhagic Complications in Primary and Secondary Thrombocytosis*

Thrombohemorrhagic complications at the time of diagnosis were found in six (23.1%)patients in PT[three (11.5%) with ischemic stroke, one (3.9 %) limb ischemia, one (3.9 %) bleeding of ruptured corpus luteum, and one (3.9 %)had intra-operative bleeding],while none of these occurred in ST patients (p = 0.009). All cases of arterial thrombosis (four patients; 15.4 %) were *JAK2 *V617F mutated ET whereas all hemorrhagic complications (two patients; 7.7%) occurred in cases of CML (Table 3). All of bleeding patients had low vWF:RCo.

*Primary and Secondary Outcomes*

The Se, Sp, PPV and NPV of abnormal LTA induced by Epi, collagen, AA and Epi plus at least one agonist of collagen and AA in the differentiation of PT from ST are 

**Table 2:** Clinical Characteristic Frequency of Patients with Primary and Secondary Thrombocytosis

	Total N = 52(100%)	PT N = 26 (100%)	ST N = 26 (100%)	P-value
Male	23 (44.2)	12 (46.2)	11 (42.3)	0.78
Anemic Symptom	3 (5.8)	1 (3.8)	2 (7.7)	1
Fever	18 (34.6)	3 (11.5)	15 (57.7)	< 0.001
B-symptom	18 (34.6)	6 (23.1)	12 (46.2)	0.08
Hepatomegaly	25 (48.1)	16 (61.5)	9 (34.6)	0.052
Splenomegaly	20 (38.5)	18 (69.2)	2 (7.7)	0.001

**Table 3:** Comparison of Frequency of Parameters such as Platelet Function Tests Using Light Transmission Platelet Aggregometry, Inflammatory Markers, von Willebrand Factor Antigens, Ristocetin Cofactor Activity and Thrombohemorrhagic Complications between Primary and Secondary Thrombocytosis

	PT (n = 26)	ST (n = 26)	P-value
	N (%)	N (%)	
Abnormal LTA			
ADP	5 (19.2)	2 (7.7)	0.223
Epi	13 (50.0)	3 (11.7)	0.003
Collagen	10 (38.5)	3 (11.6)	< 0.001
Ristocetin	7 (26.9)		0.159
AA	7 (26.9)	0	0.004
Epi and at least one: high dose ADP, collagen, high dose ristocetin, and AA	11 (42.3)	2 (7.7)	0.004
Abnormal levels of inflammatory marker			
ESR	9 (34.6)	23 (88.5)	< 0.001
CRP	10 (38.5)	21 (80.8)	0.002
ESR and CRP	5 (19.2)	18 (69.2)	< 0.001
ESR or CRP	14 (53.9)	26 (100)	< 0.001
Low vWF:Ag	2 (7.7)	0	0.149
Low vWF:RCo	11 (42.3)	3 (11.54)	0.012
Thrombohemorrhagic complications	6 (23.1)	0	0.009

PT, primary thrombocytosis; ST, secondary thrombocytosis; ADP, adenosine diphosphate; Epi, epinephrine; AA, arachidonic acid;ESR, erythrocyte sedimentation rate; CRP, C-reactive protein; vWF:Ag, von Willebrand factor antigen; vWF:RCo, ristocetin cofactor activity.

The Se, Sp, PPV, and NPV of abnormal ESR in the differentiation of ST from PT were 88.5%, 65.4%, 71.9% and 85% while the corresponding figures for abnormal CRP were 80.8%, 61.5%, 67.7% and 76.2%, respectively. The Se, Sp, PPV, and NPV of low vWF:Ag in the differentiation of PT from ST were 7.69%, 100%, 100% and 52% while low vWF:RCo were 42.3%, 88.5%, 78.6% and 60.5%, respectively (Table 5).

**Table 4:** Comparison of Median Parameters such as Platelet Function Tests Using Light Transmission Platelet Aggregometry, Inflammatory Markers, Von Willebrand Factor Antigens, Ristocetin Cofactor Activity and Thrombohemorrhagic Complications between Primary and Secondary Thrombocytosis

	PT (n = 26)	ST (n = 26)	P-Value
	median(Range)	median(Range)	
LTA			
ADP	74.5 (18 – 100)	74 (35 – 97)	0.707
Epi	60 (4 – 100)	74.5 (12 – 93)	0.012
Collagen	71 (0 – 100)	76.5 (60 – 93)	0.115
Ristocetin	80.5 (9 – 100)	82.5 (16 – 98)	0.475
AA	77 (8 – 100)	77 (62 – 99)	0.741
Inflammatory markers,			
ESR (mm/hr)	8.4 (1-67)	57 (1-79)	< 0.001
CRP (mg/L)	3.8 (3.1-108)	27.1 (3.1-194)	< 0.001
ESR and CRP	5 (19.2)	18 (69.2)	< 0.001
ESR or CRP	14 (53.9)	26 (100)	< 0.001

PT, primary thrombocytosis; ST, secondary thrombocytosis; LTA, light transmission platelet aggregometry; SD, standard deviation; ADP, adenosine diphosphate; Epi, epinephrine; AA, arachidonic acid;ESR, erythrocyte sedimentation rate; CRP, C-reactive protein; vWF:Ag, von Willebrand factor antigen; vWF:RCo, ristocetin cofactor activity.

*Subgroup Analysis in Essential Thrombocythemia Patients*

 The sensitivity, specificity, PPV and NPV of abnormal LTA induced by Epi, collagen, AA and Epi plus at least one agonist of collagen and AA in the differentiation of ET from ST are shown in Table 5. The Se of each agonist was 50%, 40%, 30%, and 30%, respectively. The specificity of Epi was 88.5% and other agonists were all 100%. PPV of Epi was 62.5% and 100% for others. The NPV of each agonist was 82.1%, 81.3%, 78.8%, 78.8%, respectively. The Se, Sp, PPV and NPV of abnormal ESR in the differentiation of ST from ET were 88.5%, 60%, 85.2% and 66.7% while the corresponding figures for abnormal CRP were 80.8%, 60%, 84% and 54.5% respectively.

*Factors Associated with Thrombohemorrhagic Complications*

The patients with thrombohemorrhagic complications had a significantly greater mean platelet count compared with patients without these complications (1,837 x 109 /L vs. 810.1x 109 /L, p < 0.001). Abnormal LTA induced by Epi (100% vs. 21.74%, p < 0.001) and collagen (66.67% vs. 13.04%, p = 0.009) presented significantly in patients with thrombohemorrhagic complications. No significant difference in the levels of ESR, CRP, vWF:Ag and vWF:RCo were noted between groups.

From the univariable analysis, risk factors associated with thrombohemorrhagic complications were a mean platelet count greater than 1,837 x 109/L (OR 1.01; 95% CI 1.002 - 1.009; p = 0.006), Abnormal LTA using Epi (OR 0.90; 95% CI 0.815 - 1.003; p = 0.057), and abnormal LTA using collagen (OR 0.97; 95% CI 0.946 – 0.998; p = 0.034). However, no significant risk factors were associated with thrombohemorrhagic complications according to the multivariable analysis.

**Table 5:** Primary Outcomes, Secondary Outcomes, and Subgroup Analysis of Outcomes in Essential Thrombocythemia

	Se ( %,) (95% CI)	Sp (%), (95% CI)	PPV (%), (95% CI)	NPV (%), (95% CI)
Primary outcomes				
Abnormal LTA for differentiation of PT from ST.				
Epi	50 (29.9-70.1)	88.5 (69.8-97.6)	81.3 (54.4-96)	63.9 (46.2-79.2)
Collagen	38.5 (20.2-59.4)	100 (86.8-100)	100 (69.2-100)	61.9 (45.6-76.4)
AA	26.9 (11.6-47.8)	100 (86.8-100)	100 (59-100)	57.8 (42.2-72.3)
Epi and either collagen or AA	38.5 (20.2-59.4)	100 (86.8-100)	100 (69.2-100)	61.9 (45.6-76.4)
Secondary outcomes				
Inflammatory markers for differentiation of PT from ST.				
High ESR	88.5 (69.8-97.6)	65.4 (44.3-82.8)	71.9 (53.3-86.3)	85 (62.1-96.8)
High CRP	80.8 (60.6-93.4)	61.5 (40.6-79.8)	67.7 (48.6-83.3)	76.2 (52.8-91.8)
High ESR and CRP	69.2 (48.2-85.7)	80.8 (60.6-93.4)	78.3 (56.3-92.5)	72.4 (52.8-87.3)
High ESR or CRP	100 (86.8-100)	46.2 (26.6-66.6)	65 (48.3-79.4)	100 (73.5-100)
vWF:Ag and vWF:RCofor differentiation of PT from ST.				
Low vWF:Ag	7.69 (0.9-25.1)	100 (86.8-100)	100 (15.8-100)	52 (37.4-66.3)
Low vWF:RCo	42.3 (23.4-63.1)	88.5 (69.8-97.6)	78.6 (49.2-95.3)	60.5 (43.4-76)
Abnormal LTA for the differentiation of ET from ST.				
Epi	50.0	88.5	62.5	82.1
Collagen	40.0	100.0	100.0	81.3
AA	30.0	100.0	100.0	78.8
Epi and either collagen or AA	30.0	100.0	100.0	78.8
Inflammatory markers for the differentiation of ET from ST.				
High ESR	88.5	60.0	85.2	66.7
High CRP	80.8	60.0	84.0	54.5
High ESR and CRP	69.2	70.0	85.7	46.7
High ESR or CRP	100.0	50.0	83.9	100.0

PT, primary thrombocytosis; ST, secondary thrombocytosis; LTA, light transmission platelet aggregometry; Se, sensitivity; Sp, specificity; PPV, positive predictive value; NPV, negative predictive value; ADP, adenosine diphosphate; Epi, epinephrine; AA, arachidonic acid; ESR, erythrocyte sedimentation rate; CRP, C-reactive protein; vWF:Ag, von Willebrand factor antigen; vWF:RCo, ristocetin cofactor activity; ET: essential thrombocythemia.

## Discussion

This was a prospective study aimed at determining the role of the platelet function test as well as inflammatory markers, vWF:Ag, and vWF:RCo in distinguishing between primary and secondary thrombocytosis. The results of this study may be helpful for differentiating the diagnosis of MPN especially ET from other form of ST. Since in cases of non-mutated *JAK2*V617F, *CALR *and myeloproliferative leukemia (*MPL*), according to the 2016 World Health Organization diagnostic criteria, the diagnosis of ET is still based on the absence of reactive thrombocytosis (Arber et al., 2016). The diagnosis of ST is straightforward in patients who have a clinically apparent underlying disorder such as autoimmune disease, concurrent infection and active malignancy (Schafer, 2004; Rose et al., 2012). However, the diagnosis is challenging in the cases of unclear causes of thrombocytosis. In such patients, signs and symptoms may be initially helpful in the differentiation between PT and ST. From this study, fever was less common in cases of PT compared with ST while splenomegaly was common in PT compared with ST. Regarding the blood counts, median leukocyte and mean platelet counts were higher in PT group compared with ST group. However, there was overlap in these clinical manifestations and blood count results between patients who have PT or ST. As a result, further laboratory tests that have a good sensitivity and specificity in distinguishing between these two conditions are warranted.

From the current study, abnormal LTA induced by Epi, collagen and AA had high specificity (88.5%, 100% and 100% respectively), but low sensitivity in the differentiation between PT and ST (50%, 38.5% and 26.9% respectively). These results correlated with a previous study which used the PFA-100 collagen-epinephrine (CEPI) cartridge test and epinephrine induced platelet aggregometry that showed high specificity (92% and 96% respectively) in the differentiation between ET and ST (Tsantes et al., 2010). In addition, this previous study also reported a high sensitivity (78.9% and 92% respectively) which might be explained by various methods of platelet function test, as well as differing plasma or buffers used in each study (Grignani et al., 2009). The finding that Epi had a higher sensitivity compared with collagen and AA was consistent with another study (Avram et al., 2001).

Abnormally high ESR and CRP had a high sensitivity (88.5% and 80.5% respectively) when it came to distinguishing between ST and PT. Moreover, the sensitivity increased up to 100% when an elevation of either ESR or CRP was used. So,a diagnosis of ST is very unlikely if both the ESR and CRP are normal. Levels of either CRP or ESR were described previously as being elevated in ST (Bashir et al., 2014; Tefferi et al., 1994). However, ESR and CRP had moderate specificity (65.4% and 61.5% respectively) to enable differentiation of PT from ST. In some reports, it had been stated that chronic inflammation may be an important driving force for clonal evolution and disease progression in the Ph- negative MPN (Hasselbalch et al., 2012; Geyer et al, 2015). The accuracy of using ESR and CRP as screening tool due to high sensitivity and using LTA as confirmatory test because of high specificity should be evaluated in the large further study.

From our current study, a low level of vWF:RCo was found in only 11% of patients with PT with a sensitivity of 42.3% but it had high specificity (88.5%) when it came to the determination of PT while low vWF:Ag was not significantly present between the two groups. These results correlated with the findings reported by theInternational Society on Thrombosis and Hemostasis (ISTH) registry that functional assays of vWF either vWF:RCo or collagen binding activity (vWF:CBA) appeared to be the most sensitive measurement of the vWF defect in acquired von Willebrand disease (vWD) including MPN (Federici et al., 2000). On the other hand, our current study showed that approximately 12% of patients with ST also had low vWF:RCo or had acquired vWD. This finding was in contrast to a previous report which showed that vWF:RCo was significantly higher among ST patients especially those with a platelet count of more than 700 x 109/L and none out of 60 ST patients developed acquired vWD (Rottenstreich et al., 2018). However this finding was in the line with another case report concerning a hepatocellular carcinoma patient who had extreme thrombocytosis with a low vWF:RCo but high vWF:Ag resulting from antibodies that interfere with the binding of vWF to platelets without causing clearance of the vWF antigen (Chen et al., 2005).

The major limitation of this study was the small sample size since the calculated number of participants of 46 was based on the estimated percentage of abnormal LTA in PT being 80% whereas it is 30% in ST. However, the result revealed an abnormal LTA with Epi of only 50% and 12% in PT and ST cases, respectively. As a result, the 50 participants enrolled onto this study might be not adequate to detect statistical differences with a power of 95%. The current study also had a limitation in the number of the patients with thrombohemorrhagic complications. However, these complications were also seen frequently in patients with PT with all cases of thrombotic complications occurring in *JAK2 *V617F mutated ET patients and all hemorrhagic complications were found in CML. This finding correlated with the incidence of major thrombosis events from a large cohort study of ET being 18% (Carobbioet al., 2008) and the incidence of hemorrhagic complications in the chronic phase of CML was 20% (Wehmeier et al., 1991). As a result, the identification of PT was important since it lead to the initiation of the appropriate therapy in the patients at high risk of thrombohemorrhagic complications.

Another limitation was the heterogeneity of patients in both the PT and ST groups. However, the subgroup analysis in the ET patients still revealed a high specificity of abnormal LTA by collagen (100%) and AA (100%) to distinguish between ET and ST as well as a high sensitivity and NPV of using either abnormal ESR and CRP (100%) for this purpose. The subgroup analysis in CML, PV, and PMF were not analyzed. Future studies including higher numbers of patients as well as more specific groups of patients need to be performed to confirm the findings reported in this study.

In conclusion, abnormal platelet function determined by LTA with collagen, AA, epinephrine had a high specificity in enabling the differentiation between PT and ST. The use of vWF:RCo, ESR and CRP levels could well be helpful in differentiating between PT and ST. PT has a higher incidence of thrombohemorrhagic complications compared to ST but no significant risk factors associated with these complications were found.
